# High-altitude hyperuricemia: a double-edged sword of adaptation and maladaptation

**DOI:** 10.3389/fpubh.2026.1835701

**Published:** 2026-07-27

**Authors:** Siyang Wang, Jie Gao, Xiangmei Chen

**Affiliations:** 1 Department of Nephrology, The First Medical Centre, Chinese PLA General Hospital, Chinese PLA Institute of Nephrology, State Key Laboratory of Kidney Diseases, National Clinical Research Center for Kidney Diseases, Beijing, China; 2 Department of Emergency Medicine, Xinqiao Hospital, Army Medical University (Third Military Medical University), Chongqing, China; 3 Department of Nephrology, Shandong Provincial Hospital Affiliated to Shandong First Medical University, Jinan, Shandong, China

**Keywords:** high altitude, hyperuricemia, metabolism, gut microbiota, kidney

## Abstract

Hyperuricemia is characterized by elevated serum uric acid levels and is associated with multiple diseases. Studies have consistently demonstrated that the prevalence of hyperuricemia in high-altitude areas is significantly higher than that in low-altitude areas. For high-altitude hyperuricemia, some environmental and genetic factors exhibit distinct characteristics among patients. Hyperuricemia also contributes to the onset and progression of a range of high-altitude-related disorders. Although studies have identified the characteristics of high-altitude hyperuricemia, the associations between elevated uric acid levels and influencing factors remain to be fully elucidated. This perspective compiles data from global epidemiological studies and clinical practices, and provides an exploration of the factors contributing to elevated uric acid levels at high altitudes. Additionally, we uncover the metabolic processes in patients with hyperuricemia and the correlation between hyperuricemia and high-altitude-related diseases, providing a foundation for developing more targeted therapeutic approaches and advanced pharmaceutical strategies.

## 1 Introduction

High-altitude environments are characterized by low atmospheric pressure and reduced oxygen partial pressure, both of which decrease with increasing elevation. At 5,000 meters above sea level, the partial pressure of oxygen is only half that observed at sea level. This induces systemic hypoxia. Hyperuricemia (HUA), defined as an elevated serum uric acid (SUA) concentration, is a known predictor for gout development. Beyond its association with numerous factors such as age, sex, place of residence, hypercholesterolemia, hypertriglyceridemia, impaired fasting glucose, hypertension, obesity, abdominal obesity, alcohol consumption, and sleep patterns, HUA also elevates the risk of stroke, heart disease, and chronic kidney disease (CKD) (1). The prevalence of HUA is escalating in China, posing a significant public health challenge. Studies indicate a substantially higher incidence of HUA in high-altitude regions compared to lowland areas (2).

The prevalence of HUA in the general Chinese population ranges from 8.0 to 13.3% (3, 4). Notably, a study of 4,198 enterprise employees residing at 2,600–3,700 m altitude in Qinghai, China, reported a significantly elevated HUA prevalence of 28.1% (5). This finding is consistent with the previously reported prevalence of 29.1% among residents aged ≥18 years in Lhasa, Tibet (approximately 3,800 m altitude) (2).

Given China’s vast high-altitude territories and their substantial resident populations, elucidating the epidemiological characteristics, precipitating factors, and risk factors for HUA in these regions is of critical clinical importance for developing effective treatment and prevention strategies specific to high-altitude environments.

## 2 Incidence and influencing factors

The risk of developing hyperuricemia is influenced by multiple factors, including genetic variations, environmental factors, gene–environment interactions, and age, sex, and body weight (6). In addition, diet, alcohol consumption, fructose intake, pharmacological interventions, and conditions such as obesity and insulin resistance can also contribute to the development of hyperuricemia (7).

**Figure 1:**
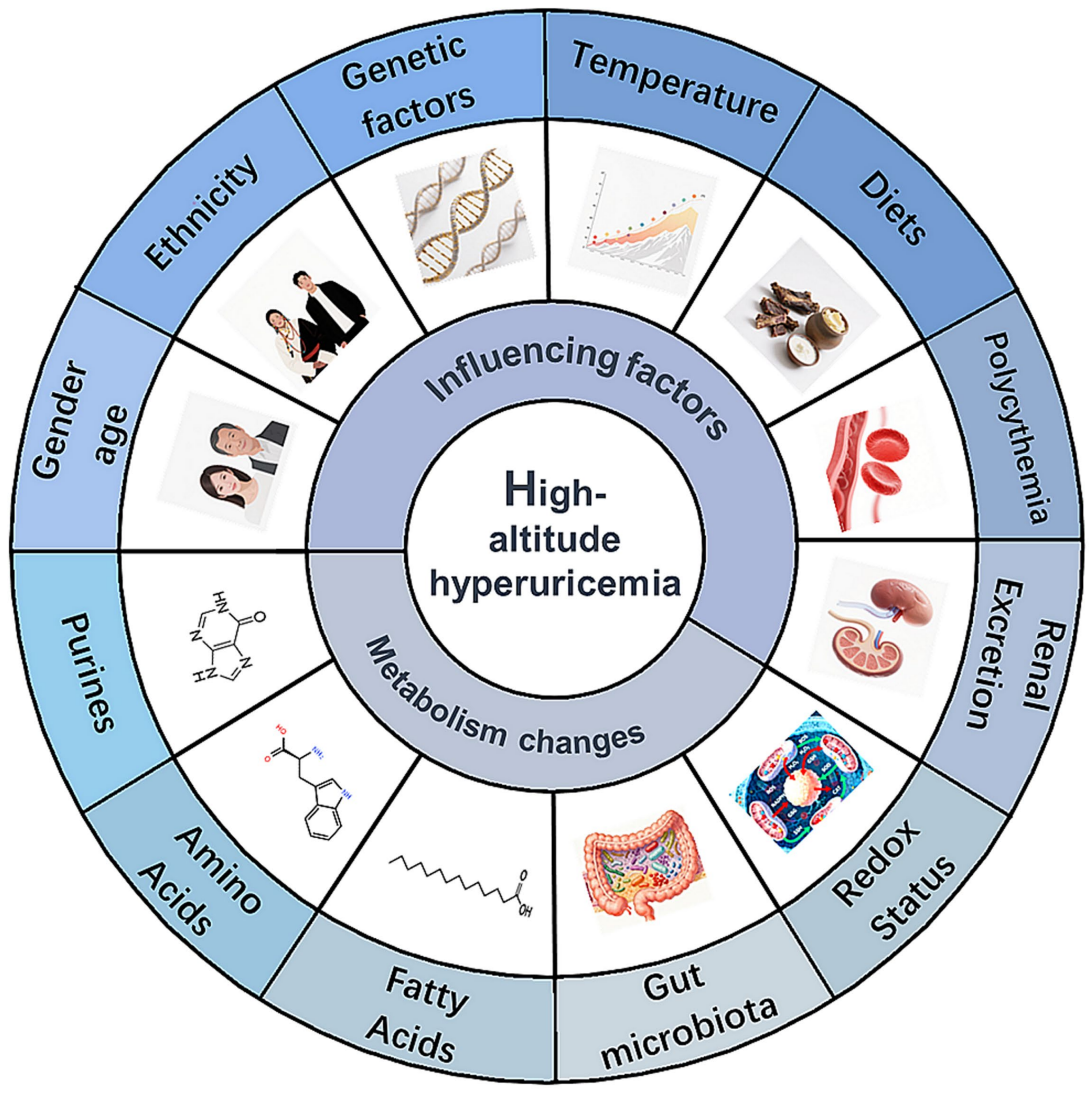
Influencing factors and metabolic changes of high-altitude hyperuricemia. Factors including gender, ethnicity, genetics, increased red blood cells, lifestyle and metabolic processes are directly involved in the development of hyperuricemia.

The factors associated with hyperuricemia at high altitudes are similar to those on low-altitude regions. Age, Body Mass Index (BMI), levels of physical activity, hypertension, diabetes, dietary patterns, and alcohol consumption are all associated with hyperuricemia. Additionally, dyslipidemia is also a risk factor for hyperuricemia (8). However, in the special environment of high altitude, some factors have a particularly notable impact on uric acid levels (Figure 1).

### 2.1 Gender

For the definition of hyperuricemia, epidemiologic studies in the United States have generally accepted in non-high altitude areas that SUA ≥ 7.0 mg/dL (416.0 μmol/L) in adult males and SUA ≥ 6.0 mg/dL (357.0 μmol/L) in adult females (9). Studies investigating uric acid levels at high altitudes has reached generally consistent conclusions regarding gender differences. HUA prevalence is higher in males than in females.

A recent cross-sectional study in the Yunnan region of China (average altitude 2,300 m) involving 1,393 Bai ethnic participants investigated the prevalence and risk factors of HUA (8). The overall HUA prevalence was 24.8%. Although lower than rates observed in high-altitude regions such as Qinghai and Lhasa, this prevalence remains significantly elevated compared to low-altitude regions. The observed associations with gender and age align with findings from other studies in China. Another cross-sectional study of over 700 Naxi ethnic individuals residing in Yunnan at approximately 2,400 m altitude reported an overall HUA prevalence of 41.31%, with prevalence rates of 57.17% in males and 16.00% in females. While this gender disparity is consistent with prior studies, the prevalence in males was substantially higher than reported in other regions; the underlying reasons for this discrepancy remain unexplained in the current literature (10).

The lower prevalence of HUA among females at high altitudes may be attributable to the suppressive effect of hypoxic conditions on white blood cell (WBC) counts. A strong correlation between high-altitude polycythemia and HUA has been established. However, one study revealed that this association is more pronounced in males, whereas females exhibit a stronger correlation with elevated WBC counts (11). By investigating the relationship between WBC subtypes and serum SUA levels, it was shown that there is a strong association between neutrophils and high SUA levels. WBC are markers of immune response and inflammation. Hypoxic environments at high altitudes often suppress immune responses, leading to a decrease in WBC counts (including neutrophils). In the normal control group, women have lower neutrophil counts than men. Therefore, the prevalence of HUA is lower in women at high altitudes. Genetic variations may be present that influence the responsiveness of red blood cell (RBC) and WBC counts to hypoxic conditions at high altitudes, potentially exerting differential effects on SUA levels between sexes. But the mechanism is still unclear. Estrogen levels may further modulate WBC production (12). Elevated plasma levels of estradiol (E2) can lead to an increase in neutrophils/monocytes. This may be the reason why female patients with HUA have a stronger association with elevated WBC counts.

### 2.2 Age

Prior to puberty, the mean SUA level is 3.6 mg/dL (214.0 μmol/L) in both males and females. After puberty, SUA levels rise to adult values, with women typically maintaining concentrations approximately 1 mg/dL lower than men (13). Following menopause, uric acid (UA) levels in men and women become similar (14). Age-related differences in SUA levels in women may be attributed to enhanced renal uric acid clearance mediated by estrogen, which diminishes after the menopausal transition (15). Furthermore, whereas the prevalence of HUA decreases gradually with advancing age in males, it increases in females aged ≥40 years, peaking between 50 and 60 years of age (8). This sex-specific variation in the age-related prevalence of HUA may be partly due to interactions with sex hormones.

### 2.3 Ethnicity

With the economic development of China, an increasing number of ethnic groups, primarily Han Chinese, have established residence in Tibet. Investigating the metabolic and physiological changes during their acclimatization to high altitude will improve our understanding of the impact of altitude on these migrant populations and potential ethnic variations. A survey among Tibetan residents aged over 40 years living at altitudes exceeding 4,500 meters revealed a remarkably low prevalence of HUA, at merely 2.05% (16).

Most existing research has focused on Han Chinese and Tibetan populations. In the present study, longitudinal biochemical data collected over three years from Han Chinese and Tibetan individuals who residing at approximately 4,300 meters for at least five years were analyzed. The findings indicated that UA levels remained relatively stable over the observation period in both ethnic groups. However, the mean UA levels among both Han Chinese and Tibetan settlers consistently exceeded the upper threshold of the established reference range. Furthermore, consistent with other studies, Tibetan individuals exhibited significantly lower UA levels compared to their Han Chinese counterparts (17).

### 2.4 Polycythemia

The association between high-altitude-induced secondary polycythemia and HUA has been recognized since 1937, with early observations indicating a strong correlation between high-altitude erythrocytosis and elevated serum urate levels (18). UA accumulation is thought to be result from to purine metabolism during erythrocyte turnover (19). Under hypoxic conditions at high altitudes, compensatory erythrocyte production accelerate hemoglobin synthesis and degradation, thereby increasing purine metabolism and UA generation.

In 2022, a comparative study involving residents of Cerro de Pasco, Peru (4,300 m) with excessive erythrocytosis (EE; hematocrit >65%) and sea-level controls further substantiated this relationship. Individuals with EE at high altitude exhibited significantly elevated serum urate levels, increased 24-h urinary urate excretion, and a higher urate-to-creatinine ratio (20). Additionally, among 672 Tibetan males (aged 18–60 years) residing at a mean altitude of 4,014 m, HUA prevalence reached 49.4%. Subgroup analysis revealed substantially higher HUA rates in polycythemic individuals than in their non-polycythemic counterparts, with UA levels showing a positive correlation with both hematocrit and hemoglobin concentrations (21).

### 2.5 Renal excretion

Impaired renal excretion of UA is also a significant contributor to elevated UA levels at high altitude. Chronic exposure to high-altitude environments induces polycythemia and elevated hematocrit, concomitant with a decrease in renal blood flow. This reduction in renal perfusion leads to a decline in glomerular filtration rate (GFR), resulting in diminished UA excretion (22). HUA is also associated with activation of the renin-angiotensin-aldosterone system (RAAS), which promotes vasoconstriction of the afferent arterioles and reduces renal plasma flow. Clinical trials have consistently demonstrated that pharmacological interventions targeting UA reduction significantly improve estimated glomerular filtration rate (eGFR) in individuals diagnosed with CKD (23).

Additionally, lactate accumulated under hypoxic conditions at high altitude enhances urate reabsorption via the organic anion exchange transporter in the proximal renal tubules. Specifically, lactate competes with urate for excretion in the proximal tubules, consequently decreasing urate clearance (24).

HUA is associated with albuminuria. Multivariate analyses have identified HUA, polycythemia, and hypertension as independent predictors of albuminuria (2). In 2011, Hurtado et al. proposed the concept of high-altitude renal syndrome (HARS), characterized by high-altitude polycythemia, HUA, systemic hypertension, and microalbuminuria (25).

Urate anion transporter 1 (URAT1) and glucose transporter 9 (GLUT9) are the primary transporters responsible for urate reabsorption, whereas organic anion transporter 1 (OAT1) and organic cation transporter 1 (OCT1) are the major transporters facilitating urate secretion. Studies revealed that rats exposed to a hypobaric hypoxia chamber exhibited significantly reduced renal expression levels of OAT1 and OCT1 compared to controls. Conversely, URAT1 and GLUT9 levels showed a slight increase, although this was not statistically significant. Therefore, it is hypothesized that under hypoxic conditions, the increase in renal UA retention is primarily attributed to reduced urate excretion, rather than enhanced reabsorption.

### 2.6 Genetic factors

A large-scale European study involving 140,000 participants demonstrated that SUA levels are significantly influenced by genetic determinants (26). Specifically, the ATP-binding cassette transporter G2 (*ABCG2*) located on chromosome 4 has been identified as a principal regulator of SUA concentrations. *ABCG2* is primarily expressed in the brush-border membrane of renal proximal tubular cells, playing a crucial role in UA excretion and the regulation of serum concentrations. Notably, functional mutations in *ABCG2* have been shown to contribute to the pathogenesis of HUA and gout (27).

Several studies have performed genotyping on single nucleotide polymorphisms (SNPs) of *ABCG2* in Chinese Tibetan patients with gout (28). Significant differences in mean SUA levels were observed across different genotypes of the *ABCG2* rs3114018 polymorphism, which is consistent with findings in Chinese Han populations (Supplementary Table S1). However, other ABCG2 polymorphisms that demonstrated significant differences in Han populations, such as rs2231142 and rs4148155, did not show significant associations in Tibetan populations (29). Another genome-wide association study investigating SNPs in Tibetan gout patients identified 11 SNPs in WD repeat-containing protein 1 (*WDR1*) and cytokine-dependent hematopoietic cell linker (*CLNK*) genes (30). This study revealed associations between specific *CLNK* SNP genotypes and higher average serum glucose (GLU) levels, serum high-density lipoprotein cholesterol (HDL-C) levels and SUA levels. Polymorphisms in the *ABCG2* and polycystin 2 (*PKD2*) genes influence multiple risk factors associated with gout progression. PKD2, expressed in the cell membranes of the distal renal tubules and collecting ducts, are associated with an increased risk of gout in the Tibetan population (31). Genetic variations are likely to influence whether the increase UA stays within an adaptive range or enters a pathogenic range. This balance is affected by metabolic changes caused by hypoxia.

### 2.7 Other influencing factors

Research on the impact of temperature on UA levels remains limited. Comparing daily mean temperatures across altitudes ranging from 1,400 m to 4,400 m in northwestern China, one study reported that a 5 °C decrease in mean temperature was associated with increases of 5.07% (95% CI: 3.52, 6.63%) in triglyceride (TG) levels and 2.85% (95% CI: 2.18, 3.53%) in UA levels, respectively (32). This study identified a negative association between temperature and UA levels. Cold exposure may elevate serum lipid concentrations, potentially promoting lipid deposition. This process could further enhance purine transport, and since UA is the final metabolite of purine catabolism, its production could increase. Concurrently, hyperlipidemia may impede renal UA excretion, thereby reducing clearance and ultimately elevating SUA concentrations (33).

Owing to its unique geographical environment, Tibet exhibits distinctive ethnic dietary characteristics. The Tibetan diet is typified by a high consumption of meats, primarily derived from sheep, cattle, pigs, and poultry, with common cooking methods including roasting, frying, and boiling. Consequently, this dietary pattern is characteristically high in fat and protein. Such diets, particularly those rich in purines or fructose, are known to elevate SUA levels. Furthermore, the consumption of highland barley wine among high-altitude residents may also be a contributing factor to elevated UA levels. Choi et al. analyzed data from the Third National Health and Nutrition Examination Survey (NHANES III) in the United States and found that SUA levels increased with higher intake of beer and alcoholic beverages (34). A study in China also supported that alcoholic beverage was associated with increased SUA. Data from the China National Health Survey (CNHS) among adults from 10 provinces in China revealed that alcohol consumption, particularly higher daily alcohol consumption, has a more significant impact on SUA levels (35).

## 3 High-altitude hyperuricemia and metabolism

The impact of high altitude on metabolism and its role in the human response to environmental changes have also attracted considerable attention. However, due to the complex interplay among genetic, environmental, and lifestyle factors, the underlying biological processes regulating SUA levels and metabolism remain incompletely characterized. Numerous studies employing metabolomics and proteomics approaches have elucidated the influence of metabolic on high-altitude HUA, implicating a link between metabolic alterations and HUA. Chinese Han individuals were acclimated at altitudes of 4,500 m and 5,300 m for a period from 6 months to 1 year. Proteomic analysis of serum samples was conducted to compare the high-altitude group with the sea-level group. Differentially expressed proteins (DEPs) were identified, including 20 significantly upregulated and 34 significantly downregulated proteins. These DEPs are implicated in various metabolic pathways, particularly those related to hypoxic cellular metabolism (36). Human serum metabolomics analysis has also identified pathways and metabolites that are crucial for regulating serum urate levels at various altitudes (37, 38). This reflects an important correlation between metabolism and high-altitude HUA.

### 3.1 Metabolism of purines, amino acids and fatty acids

The elevation in SUA levels observed at high altitudes may also be associated with increased adenine nucleotide turnover, activation of xanthine oxidase (XOD), and reduced adenosine triphosphate (ATP) levels. Under hypoxic conditions, ATP is degraded into adenosine monophosphate (AMP). Subsequently, purine bases are catabolized further to adenosine, inosine, and hypoxanthine. Xanthine dehydrogenase, the rate-limiting enzyme in purine nucleotide degradation, catalyzes the final steps in UA production. Notably, XOD activity increases under ischemic/hypoxic conditions, where it oxidizes hypoxanthine to xanthine and ultimately to UA, while generating superoxide free radicals (39). Moreover, adenosine deaminase (ADA), a crucial enzyme in purine nucleoside metabolism, catalyzes the deamination of adenosine to inosine. Inosine is subsequently metabolized to hypoxanthine, which is a substrate for XOD-mediated oxidation to UA.

In healthy individuals, exposure to high altitudes for a short period can result in changes in various amino acids and purines (40, 41). A study investigated the effects of 12-month exposure at an average altitude of 2,900 m on serum urate levels and the metabolome in 49 healthy Han Chinese individuals originating from low-altitude regions (<50 m). A local cohort of 47 healthy long-term residents (≥5 years) in Linzhi (comprising 36 Han Chinese and 11 Tibetans) was concurrently enrolled to assess the potential of duration of moderate-altitude exposure on serum urate and the metabolome (42). Compared with baseline levels, serum urate concentrations in the 12-month exposure group exhibited an increasing trend, although this increase was not significant. In contrast, both Han Chinese and Tibetan long-term residents in Linzhi had significantly lower serum urate levels than the 12-month exposure group. Conversely, individuals with long-term high-altitude residence (>5 years; both ethnic groups) exhibited significantly lower SUA levels than those exposed for only 12 months, indicating potential metabolic reprogramming as an adaptive mechanism to the high-altitude environment. Notably, *β*-alanine concentration showed a significant negative correlation with SUA. Long-term residents demonstrated higher circulating *β*-alanine levels. β-Alanine, a non-essential amino acid synthesized in the liver, has been shown to inhibit XOD activity and reduce UA synthesis in hypoxanthine (HX)-induced HUA models. Given its safety profile, *β*-alanine supplementation represents a potential adjunctive therapy for HUA, which could enhance treatment efficacy while mitigating drug-related toxicity. Further investigation is warranted to determine whether β-alanine offers superior anti-inflammatory effects compared to allopurinol. A combined prediction model for asymptomatic hyperuricemia (AHU) risk was developed by integrating the differential metabolites identified in this study with phenotypic data. This model achieved a significantly improved area under the curve (AUC) of 0.93, offering a novel tool for AHU risk stratification in populations residing at moderate altitudes.

Blood samples were collected from individuals residing at altitudes of 4,300–4,500 m in China, comprising subjects with HUA (SUA > 420 μmol/L) and healthy controls, for metabolomic and proteomic analyses. Metabolomic profiling identified 79 significantly altered metabolites in the patient group. Alterations in specific pathways, notably glycerophospholipid and riboflavin metabolism, were implicated in HUA pathogenesis. Decreased levels of L-cystathionine and serine demonstrated high specificity, suggesting their potential as diagnostic biomarkers or therapeutic targets. Random forest machine learning analysis of the metabolomic and proteomic data identified 30 significant molecular features. Among these, cytosine and UA were key metabolite features, alongside 11 characteristic proteins including IGLC2, IGLC7, IGLC3, C4BPA, C1QB, and C1QC. The model exhibited outstanding performance, achieving an AUC of 1 (43). These signature metabolites and proteins show promise for the early diagnosis of HUA.

UA is closely associated with fatty acid metabolism. Studies have found that uric acid enhances the expression of lipogenic enzymes, leading to free fatty acid (FFA) accumulation and impaired energy metabolism. This may be related to the endoplasmic reticulum stress (44) and inflammasome pathways (45). Furthermore, HUA can stimulate aldose reductase expression, resulting in increased triglyceride accumulation. This stimulatory mechanism is mediated by uric acid-induced oxidative stress and the activation of nuclear factor of activated T cells 5 (NFAT5) (46). Basic research indicates that the hypoxic environment induced by high altitude disrupts fatty acid metabolism in rat kidneys, leading to lipid accumulation. This lipid accumulation subsequently induces oxidative stress and inflammatory injury to the renal tissue. Dioscin (DIO) and its active metabolite, diosgenin (DG), have been shown to modulate lipid metabolism in mice and exert UA-lowering effects. This therapeutic effect against high-altitude HUA is mediated by DG’s regulation of renal fatty acid metabolism through targeting the epoxide hydrolase 2 (*EPHX2*) gene (47).

### 3.2 Gut microbiota

Given that approximately 30% of UA is excreted via the intestines, intestinal UA metabolism represents a significant factor influencing SUA levels under hypoxic conditions at high altitudes. Furthermore, long-term high-altitude residents exhibited an altered gut microbiota composition characterized by an increase in UA-degrading bacteria (e.g., *Alistipes putredinis*) and a decrease in UA-producing bacteria (e.g., *Klebsiella pneumoniae*), suggesting a potential contribution to SUA homeostasis (42). In studies of Han and Tibetan populations residing on the Qinghai-Tibet Plateau (altitude >3,000 m), Tibetans exhibited greater overall gut microbial evenness than Han individuals. The gut microbial community structure of Tibetans was relatively more stable, and demonstrated greater functional diversity than that of Han individuals. Furthermore, Tibetans showed higher richness and diversity indices in their gut microbiota composition. At the genus level, Bacteroides predominated in the Han population, whereas *Prevotella* was the dominant genus in the Tibetan population (48).

The gut microbiota regulates intestinal UA levels through various mechanisms, including the modulation of host metabolic pathways and immune responses, the promotion of gut health, and the facilitation of UA excretion. Gut bacteria enhance the breakdown of UA, thereby reducing its systemic concentration. A cohort study observing individuals transitioning from 243 m to 3,658 m altitude revealed a significant increase in intestinal UA levels within the first 20 days following high-altitude arrival. However, intestinal UA levels subsequently displayed a declining trend over time. Furthermore, both Han and Tibetan populations residing long-term at 4,700 m in Tibet exhibited markedly lower intestinal UA levels. This suggests that reduced intestinal UA levels may contribute to adaptation to high-altitude hypoxia. Fecal metagenomics analysis of key UA-degrading bacteria, primarily from the *Lachnospiraceae* family, demonstrated consistently high abundance of these bacteria in the long-term resident cohort (49). This finding underscores their significant role in maintaining low intestinal UA levels and highlights the importance of UA homeostasis in high-altitude adaptation. When Han individuals ascend to plateaus regions exceeding 4,000 m, their gut microbial structure gradually shifts to resemble that of Tibetans with prolonged residence (50). This microbial restructuring may facilitate Han individuals’ adaptation to high altitude. Like the Tibetan population, the Han population exhibited an increase in the anaerobic to aerobic bacteria ratio during adaptation, which may confer a competitive advantage.

Modulation of the gut microbiota represents a potential strategy to mitigate hypoxia-induced injuries at high altitude. Fecal microbiota transplantation from plateau zokors (a small mammal native to the Qinghai-Tibet Plateau at ~3,500 m elevation) was performed in Sprague–Dawley (SD) rats over a 30-day period. This procedure resulted in significant alterations to the rats’ gut microbiome and pulmonary metabolism, reducing the production of pro-inflammatory mediators, including histamine and UA. Consequently, this intervention ameliorated pulmonary hypertension and enhanced their high-altitude adaptation (51).

### 3.3 Oxidative stress in metabolic process

Oxidative stress is the imbalance between the production of free radicals and reactive metabolites, such as reactive oxygen species (ROS), and antioxidants. This imbalance can lead to cell injury. Urate is the most abundant water-soluble antioxidant, accounting for approximately two-thirds of the total antioxidant capacity in human blood (52). Elevated SUA caused by short-term exposure to moderate altitudes is associated with oxidative stress (decreased superoxide dismutase/glutathione peroxidase levels) (42). An Indian study investigated differences in antioxidant and UA levels between lowland and highland residents (3,800 m-4000 m) following four weeks of chronic hypoxic exposure at 4560 m. Blood tests revealed that lowlanders exhibited significant increases in antioxidant levels, oxidative stress, and SUA concentrations after one month of high-altitude exposure. Concurrently, serum vitamin C concentrations decreased in these individuals relative to low-altitude controls, attributable to elevated serum free radical levels induced by high altitude. In contrast, highlanders demonstrated lower levels of these parameters compared to lowlanders (53), indicating better adaptation to altitude changes. Thus, in addition to the metabolic changes induced by hypoxia, the increased oxidative stress response contributes to elevated UA levels during short-term high-altitude exposure. Lowland volunteers showed significant increases in total antioxidant status, plasma antioxidant capacity, and serum urate concentrations within one week after arrival at high altitude. A highly significant correlation was observed between plasma urate concentrations and plasma antioxidant capacity (54). However, urate production is catalyzed by xanthine oxidase, a reaction inherently associated with the release of ROS (55). Consequently, excessive urate can also stimulate oxidative stress in various cell types, leading to endothelial dysfunction, systemic hypertension, and glomerular hypertension (56).

Rats were maintained in a hypobaric chamber simulating a hypoxic environment for 30 days. By day 30, Na^+^, K^+^-ATPase activity in the experimental group was significantly reduced compared to that in the control group. The reduction in Na^+^, K^+^-ATPase activity under hypoxic conditions may be associated with impaired GFR and renal tubular reabsorption capacity (57). Utilizing a 9% oxygen concentration to simulate hypoxia at altitudes exceeding 5,000 m, Poonit et al. (58) observed alterations in renal filtration function in rats, manifested by significantly increased SUA, creatinine, and blood urea nitrogen. Furthermore, chronic intermittent hypoxia (CIH) resulted in a significant downregulation of antioxidant indicators expression alongside a significant increase in SUA levels (59). These findings collectively demonstrate that hypoxia impacts systemic redox balance and stimulates inflammatory responses, suggesting its potential involvement in subsequent gout development. Additionally, significant increases in XOD and ADA activity were detected in blood.

## 4 Hyperuricemia and high altitude-related diseases

High-altitude-induced HUA is accompanied by systemic stress and inflammatory states, which makes high-altitude HUA relevant to other high-altitude-related diseases as well (Table 1).

**Table 1:** High altitude-related diseases associated with hyperuricemia.

Diseases	Symptoms	Mechanisms related to hyperuricemia
Acute mountain sickness	headache, fatigue, anorexia, nausea, or vomiting	adverse effects of elevated serum uric acid levels, such as endothelial dysfunction and mitochondrial damage
High-altitude pulmonary edema	fever, tachycardia or bradycardia, chest tightness, dyspnea, cough and expectoration	A significant elevation in SUA levels was observed.
Pulmonary hypertension and pulmonary embolism	hypoxic pulmonary vasoconstriction, increased pulmonary vascular resistance, and vascular remodeling	Elevated serum uric acid levels increase oxidative stress, which contributes to vascular endothelial injury and dysfunction, and is associated with the activation of platelets and the coagulation cascade
Low birth weight	inverse linear relationship between maternal SUA levels and birth weight, particularly in female neonates	Uric acid -induced placental dysfunction, which impairs amino acid transport within the placental system, potentially leading to intrauterine growth restriction.

### 4.1 Acute mountain sickness

The question of whether high-altitude-induced changes in clinical characteristics and blood markers correlate with acute mountain sickness (AMS) remains unclear in current literature. Symptoms of AMS typically include headache, fatigue, anorexia, nausea, or vomiting, and their severity can vary. In 2023, a study investigating alterations in clinical and biochemical responses to high altitude in 22 subjects who ascended to Lhasa, China (3,648 m), aiming to identify potential biomarkers for AMS (60). Data analysis collected two days after rapid ascent demonstrated that following exposure to 3,648 m, SUA levels increased by 17.58 ± 38.213 μmol/L in the AMS group but decreased by 40.3 ± 29.447 μmol/L in the non-AMS group. The AUC for high-altitude-induced UA changes was 0.883 (95% CI: 0.738–1.000). The specificity and sensitivity of UA change for AMS identification were 83.30% (95% CI: 51.59–97.91%) and 90.00% (95% CI: 55.50–99.75%), respectively. Individuals without AMS exhibited higher baseline UA levels both before and after acute high-altitude exposure compared to those who developed AMS. This observation may be attributable to UA’s role as the end product of human purine metabolism, which can enhance SOD activity and antioxidant effects. The reduction in UA levels following acute exposure, as opposed to a further increase, suggests an enhanced capacity for UA homeostasis maintenance in non-AMS individuals. This regulatory ability may help mitigate the adverse effects associated with elevated UA levels, such as endothelial dysfunction (61) and mitochondrial damage (62). However, the limited sample size poses a significant limitation to the generalizability of these findings.

### 4.2 High-altitude pulmonary edema

High-altitude pulmonary edema (HAPE) can present with fever, tachycardia or bradycardia, chest tightness, or dyspnea. Chief complaints include cough and expectoration. A retrospective study of 48 cases of HAPE occurring between 2,800 m and 3,000 m revealed that 96.9% of the patients were male. Simultaneously, a significant elevation in SUA levels was observed (63). Mitochondrial dysfunction induced by hypobaric hypoxia may be associated with the suppression of estrogen-related receptor *α* (ERRα) (64). ERRα is a key regulator of mitochondrial biogenesis and oxidative phosphorylation. Differential transduction of the ERRα signaling pathway between males and females at high altitudes potentially contributes to the heightened male susceptibility to HAPE.

In another study involving 429 patients (aged 18 to 75 years) diagnosed with HAPE or high-altitude cerebral edema (HACE) following ascent to altitudes exceeding 3,000 m, SUA levels were markedly elevated in HAPE patients. Furthermore, UA levels in HACE patients exceeded those observed in HAPE patients. Males accounted for 90.21% of the HAPE cases. Among the female HAPE patients, over 50% were postmenopausal (65). Given that estradiol has been demonstrated to attenuate hypoxia-induced pulmonary vasoconstriction, this mechanism may be one factor contributing to the relative protection observed in females (66).

### 4.3 Pulmonary hypertension and pulmonary embolism

High-altitude pulmonary hypertension (HAPH) affects individuals residing above 2,500 m. Its reported prevalence in the Tibetan population of China is 3.2% (67). The pathogenesis of HAPH is complex and involves hypoxic pulmonary vasoconstriction, increased pulmonary vascular resistance, and vascular remodeling. Among Tibetans residing in Lhasa, Tibet (average altitude: 4,380 m) for at least five years, 57.5% exhibited microalbuminuria. Compared to those without microalbuminuria, the microalbuminuria group demonstrated significantly elevated values for age, pulmonary artery systolic pressure, systolic blood pressure, diastolic blood pressure, hemoglobin concentration, glycated hemoglobin, serum creatinine, and SUA (68).

A retrospective study of pulmonary embolism (PE) patients occurring at approximately 5,000 m revealed that the Han Chinese individuals accounted for 82.4% of cases (69). Abnormal SUA metabolism may be a potential underlying trigger for the increased incidence of PE at high altitudes. A large Chinese cohort study on PE identified elevated SUA as a risk factor for venous thrombosis (70). Furthermore, other studies have indicated a positive correlation between elevated SUA levels and the risk of venous thromboembolic disease (71). Elevated SUA levels increase oxidative stress, which contributes to vascular endothelial injury and dysfunction, and is associated with the activation of platelets and the coagulation cascade. Additionally, the production of inflammatory cytokine in gout can activate cytokine expression and promote thrombus formation (7).

### 4.4 Other high altitude-related diseases

Low birth weight significantly affects neonatal survival rates in high-altitude regions (72). A study of 760 maternal-fetal pairs at 4,100 m altitude in China demonstrated an inverse linear relationship between maternal SUA levels and birth weight, particularly in female neonates. This association may be related to UA-induced placental dysfunction, which impairs amino acid transport within the placental system, potentially leading to intrauterine growth restriction (73). Furthermore, the incidence of low birth weight was significantly higher in female infants than in males. Interestingly, in pregnant women with proteinuria, SUA levels exhibited a U-shaped relationship with male birth weight (74).

The Bogalusa Heart Study demonstrated an association between higher childhood UA levels and an increased incidence of hypertension in adulthood (75). Similarly, measurements of multiple physiological and biochemical parameters among indigenous Argentinian children residing at 2,000–4,000 m revealed a correlation between elevated UA levels and increased blood pressure (76). This investigation defined prehypertension (preHTN) as BP values ≥90th percentile but <95th percentile, and hypertension (HTN) as BP values ≥95th percentile, according to age-, sex-, and height-specific percentiles. UA levels increased progressively across BP categories, rising from normotension to preHTN and then to HTN. However, whether this association is determined by environmental or genetic factors remains to be fully elucidated.

## 5 Monitoring and treatment

Research is actively exploring more convenient and cost-effective detection methods to facilitate effective early screening and health monitoring in high-altitude regions. Point-of-Care Testing (POCT) devices are popular due to their compact size, portability, ease of use, and rapid results. However, high-altitude environments present significant challenges for blood-based sensors, as blood viscosity, hemoglobin concentration, and hematocrit increase with elevation. Elevated hematocrit reduces the serum volume directly available for the target reaction, potentially introducing bias into assay results. Furthermore, for electrochemical sensors, increased viscosity and hematocrit severely impede electron transfer kinetics, compromising the accuracy of electrochemical sensors. To address these challenges, Yu et al. developed a simple but innovative paper-based biosensor for hemoglobin detection. They also modified a prior paper-based photochemical sensing platform to create POC devices for detecting blood lipids and UA specifically validated for high-altitude testing, demonstrating high accuracy (77).

There is currently no widely accepted therapeutic consensus specifically tailored to the management of HUA in high-altitude environments. Treatment predominantly relies on conventional low-altitude drug protocols. Drug selection must be guided by the underlying etiology, comorbidities, and hepatic and renal function status. The basic principle of urate-lowering therapy is to start with conservative dosage while preventive treatment (78). In China, the primary urate-lowering drugs used clinically are classified into two categories: UA synthesis inhibitors and UA excretion promoters. Since approximately 90% of patients with primary HUA exhibit the UA underexcretion, excretion-promoting drugs may be more broadly applicable in this population (79). XOD plays a central role in purine metabolism by catalyzing the conversion of purine metabolites to UA. Therefore, XOD inhibitors are the main drugs for UA synthesis inhibitors (80). Pharmacotherapy options as first-line treatments include allopurinol, febuxostat, topiroxostat (81). UA excretion promoters are considered second-line treatments for hyperuricemia, such as benzbromarone (82, 83) and probenecid (84, 85). At the same time, many new therapeutic drugs for hyperuricemia are currently in different stages of clinical development (7).

A unique therapeutic approach involves therapeutic apheresis, specifically therapeutic erythrocytapheresis. Clinical data from over 600 patients with high-altitude polycythemia demonstrate that this technique effectively reduces elevated erythrocyte levels, thereby decreasing blood viscosity. Consequently, it significantly reduced hemoglobin and SUA levels while increasing the eGFR (86). Furthermore, multiple treatment sessions produce superior outcomes compared to a single session.

Vitamin C, an antioxidant, mitigates cellular damage induced by free radicals, thereby reducing SUA production (87). Additionally, it competitively inhibits the anion-exchange transport system in the proximal renal tubules, leading to an increased fractional excretion of UA (88). Furthermore, Vitamin C enhances the GFR by alleviating glomerular microvascular ischemia and promoting dilation of afferent arterioles (89). Vitamin E, a potent lipid-soluble antioxidant, augments antioxidant enzyme activity and suppresses XOD-induced urate synthesis (90). In a preliminary prospective controlled study, young male recruits newly arriving at the Qinghai-Tibet Plateau (3,700 m altitude) received oral Vitamin C (500 mg/day) for one month, compared with an untreated control group received no intervention. SUA, creatinine, and blood urea nitrogen levels were measured at baseline and after one month. A separate study compared 500 mg/day Vitamin C with 75 IU/day Vitamin E to evaluate their effects on high-altitude HUA. Results demonstrated that both Vitamin C and Vitamin E significantly reduced UA levels and the incidence of HUA. Although Vitamin E supplementation resulted in lower SUA levels than Vitamin C, the difference was not significant. Vitamin C supplementation exhibited a favorable safety profile, whereas Vitamin E carries a risk of bleeding due to its anticoagulant properties—particularly with concomitant use of warfarin. Vitamin E supplementation is also contraindicated during chemotherapy and radiotherapy. Long-term high-dose use may cause nausea, diarrhea, and visual disturbances. Consequently, Vitamin C is safer than Vitamin E (91). These findings suggest that oral Vitamin C supplementation may serve as a safe, effective, and cost-effective intervention for preventing high-altitude HUA.

At high altitudes, inosine monophosphate (IMP) biosynthesis within purine metabolism is enhanced in both Han Chinese and Tibetan short-term residents living above 4,300 meters, while purine degradation is inhibited. IMP serves as one of the precursors for inosine synthesis. Plasma inosine, a purine nucleoside, is elevated under hypoxic conditions and exerts significant cytoprotective effects. Furthermore, it functions as a more efficient energy source than an equivalent amount of glucose (92). The elevated inosine levels observed at high altitude are crucial for mitigating hypoxia and supplying energy, thereby suggesting inosine as a potential therapeutic target for high-altitude adaptation.

Altitude-specific management strategies are important. For populations preparing to ascend, staged ascent protocols are recommended to allow gradual acclimatization and attenuate the hypoxia-driven surge in UA, while prophylactic vitamin C supplementation may serve as a safe and low-cost intervention with demonstrated efficacy in reducing SUA levels and the incidence of hyperuricemia. At high-altitudes, adequate hydration is essential to maintain renal perfusion and promote UA excretion, and dietary advice prioritizes locally available high-fiber staple foods while limiting purine-rich meats and alcoholic beverages. For long-term migrants and workers residing permanently at altitude, periodic monitoring of renal function is advised as a practical tool for early detection of renal impairment. Pharmacotherapy with xanthine oxidase inhibitors or uricosuric agents is reserved for patients with confirmed gout, renal impairment, or persistently severe hyperuricemia that does not respond to lifestyle measures.

## 6 High-risk populations and public health implications

Migrants moving from low to high altitudes constitute perhaps the most vulnerable population for HUA. Among 7,070 immigrants aged 15–45 years living on the Tibetan Plateau, the prevalence of HUA was 54.2% (93). In a more recent study of 284 plateau migrants in Ritu County, the prevalence of HUA reached 78.87%, with gender, age, RBC, and creatinine independently associated (94). Among 179 Han male immigrants on the Qinghai–Tibet Plateau, the overall prevalence was 40.8%, and multivariate analysis revealed that altitude exposure time and altitude exposure form were significant risk factors (95). These data consistently indicate that Han migrants are substantially more susceptible to HUA than long-term Tibetan residents. High-altitude workers, such as miners, represent another under-recognized high-risk group. A comparative study of coal mine workers found that those at high-altitude mining sites (>3,900 m) had significantly higher serum uric acid, total cholesterol, and creatine kinase than their low-altitude counterparts (96). Integrated health management models for high-altitude workers that combine periodic monitoring, dietary guidance, and health education may decrease SUA levels. The impact of maternal HUA on fetal outcomes at high altitudes is an important but understudied concern. In a study of 760 maternal-fetal pairs at approximately 4,100 m in Naqu, Tibet, maternal SUA showed a significant negative correlation with fetal birth weight, particularly in women with proteinuria, female fetuses, and primiparas (74). It suggests that SUA monitoring should be integrated into antenatal care protocols in high-altitude obstetric settings to identify pregnancies at risk for intrauterine growth restriction.

Health management models combining periodic monitoring, dietary counseling, health education, and behavioral interventions are necessary for SUA control and health literacy. Community-based health education programs have also been advocated to address risk factors such as high-fat diets, obesity, and smoking in high-altitude populations. Systematic integration of HUA prevention into public health management in high-altitude regions is urgently warranted. Given the vast high-altitude territories and large exposed populations in China, Peru, and other nations, such programs could substantially reduce the long-term burden of gout, hypertension, kidney disease, and adverse perinatal outcomes attributable to high-altitude HUA.

## 7 Prospect and challenges

**Figure 2:**
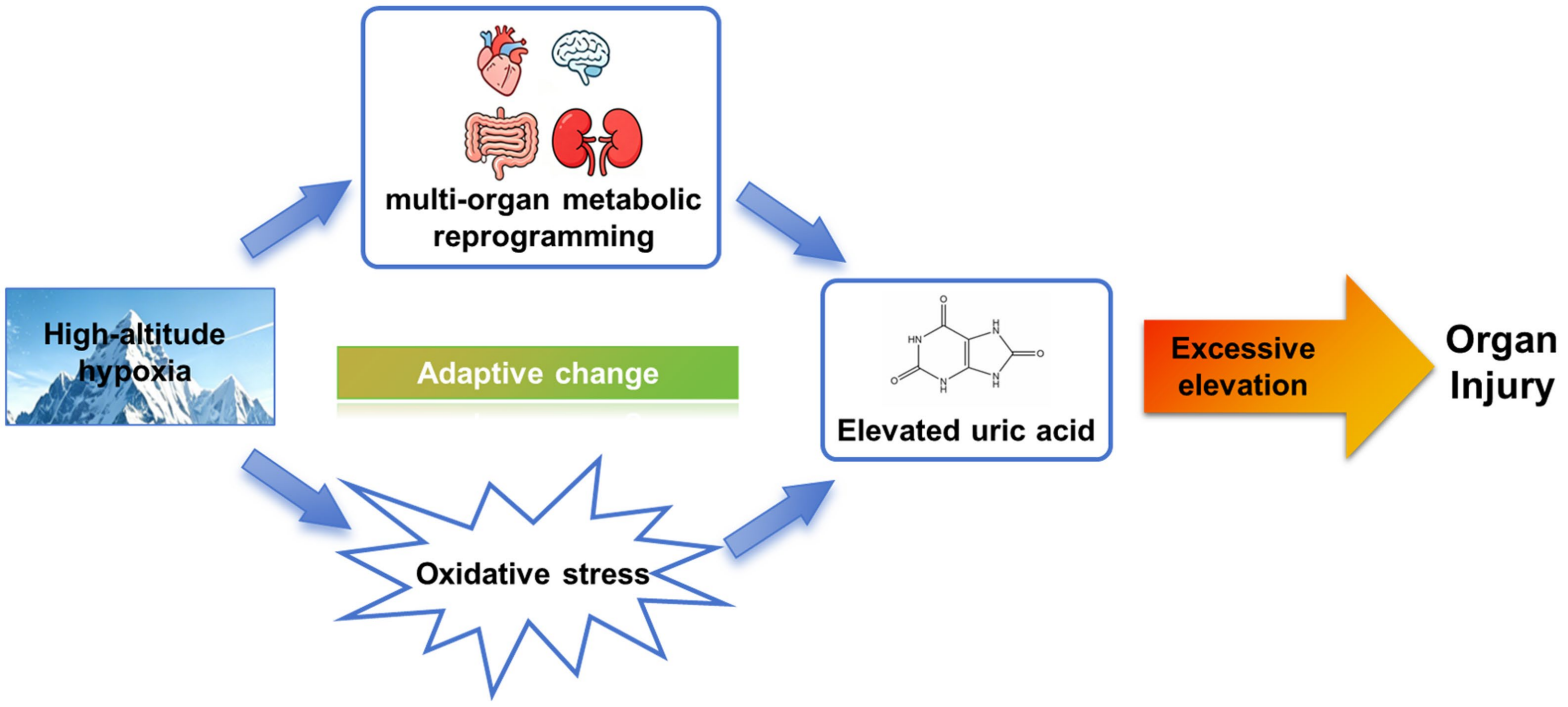
High-altitude hyperuricemia is a double-edged sword. High-altitude environments, through hypoxia-driven multi-organ metabolic reprogramming, lead to significantly elevated UA levels. This elevation simultaneously possesses the dual attributes of injury and adaptive protection.

High-altitude environments, through hypoxia-driven multi-organ metabolic reprogramming, lead to significantly elevated UA levels. This elevation simultaneously possesses the dual attributes of injury and adaptive protection (Figure 2). HUA has become increasingly recognized as a significant risk factor for a multitude of diseases. The high-altitude environment, characterized by hypobaric hypoxia, specific dietary habits, lifestyle factors, and other influences, contributes to elevated UA levels, posing a substantial health threat to populations residing at high altitudes. Factors including gender, ethnicity, genetics, and metabolic processes are directly involved in the development of HUA. Although some factors associated with HUA at high altitudes are similar to those on low-altitude regions, there are several predominant factors that are specifically associated with high-altitude HUA such as polycythemia, genetic variations, renal excretion of UA and dietary habits. Furthermore, increased SUA levels significantly impact the occurrence and development of common high-altitude pathologies such as AMS, HAPE and PE. However, the normal range of SUA in plateau environments has not yet been established with a recognized standard. And current therapeutic strategies for high-altitude HUA remain limited, primarily relying on conventional treatments and targeted erythrocytapheresis, with a notable absence of widely accepted, specifically targeted therapies. To facilitate critical appraisal of the evidence in this review, we classified the principal findings into three tiers based on study design, sample size, consistency across studies, and risk of bias (Supplementary Table S2). This classification may serve as a guide for future research.

It remains inconclusive whether the reference range for SUA requires adjustment in high-altitude environments. Substantial variations in altitude and divergent living conditions among populations residing at comparable altitudes can influence SUA levels, making it a complex issue to set different detection ranges for different situations. Currently, the reference values for SUA in high-altitude regions still follow the same standards as those established for low-altitude areas. The hypoxic environment of high altitude induces a physiological increase in SUA, which serves as a critical endogenous antioxidant. This adaptive rise is particularly pronounced during the early phase of acclimatization and is influenced by sex hormones, erythrocyte turnover, and renal hemodynamics. Consequently, many otherwise healthy high-altitude residents exhibit SUA levels that would be classified as hyperuricemia by lowland standards, yet may not carry the same pathogenic implications. Furthermore, native Tibetans consistently display significantly lower SUA levels than Han Chinese migrants living at identical altitudes. This suggests that genetic adaptation has reset the UA homeostatic set point in long-term high-altitude populations. Finally, the relationship between SUA and adverse outcomes at altitude is likely confounded by polycythemia, which itself is a strong driver of purine turnover and SUA elevation. Therefore, the pathogenic threshold for SUA-mediated tissue injury may differ between individuals with and without excessive erythrocytosis.

Currently, no prospective studies have systematically evaluated the association between altitude-specific SUA cutoffs and hard clinical endpoints in high-altitude populations, such as incident gout, progression to chronic kidney disease, cardiovascular events, or adverse pregnancy outcomes. Any recommendation to formally adjust reference ranges would therefore be premature. Yet simply applying lowland cutoffs without adjustment carries its own risks, potentially leading to both overdiagnosis and missed cases. Overdiagnosis may subject otherwise healthy individuals with adaptive hyperuricemia to unnecessary pharmacological treatment, while underdiagnosis could overlook patients in whom mildly elevated SUA signals underlying renal or vascular pathology that warrants intervention.

Resolving this dilemma requires a concerted research effort. It is necessary to establish large-scale prospective cohorts spanning multiple altitude bands, ethnic groups, and both sexes, with longitudinal tracking of SUA against incident clinical outcomes. Existing clinical laboratory databases from high-altitude hospitals represent a rich and currently underutilized resource that could be mined for preliminary reference intervals. Until more robust evidence is available, clinicians practicing at high altitudes should not rely solely on lowland cutoffs but should instead interpret SUA values within the broader context of comorbidities, acclimatization status, and concurrent markers of organ damage.

Therefore, elucidating the underlying mechanisms and key influencing factors in the pathogenesis of high-altitude HUA, along with the development of novel diagnostic and therapeutic approaches specifically tailored to the unique high-altitude environment, represent critical and promising avenues for future research.

## Data Availability

The original contributions presented in the study are included in the article/Supplementary material, further inquiries can be directed to the corresponding authors.
