# Supplementary material for: High-altitude hyperuricemia: a double-edged sword of adaptation and maladaptation
Source: Front Public Health. 2026 Jul 27;14:1835701. doi: 10.3389/fpubh.2026.1835701 (PMC13454466; doi:10.3389/fpubh.2026.1835701)
Supplement: Supplementary file 1 [file table_1.docx]

**Table S1. Characteristics of ABCG2 gene polymorphisms associated with high-altitude HUA**

| **ABCG2 SNP** | **Significance after correction** | **Key conclusion** |
| --- | --- | --- |
| rs3114018 | Han: highly significant  Tibetan: significant | The only SNP significantly associated with SUA in both Han and Tibetan |
| rs2728106  rs2728133 | Tibetan: significant | Strongly associated locus in Tibetan |
| rs17731799  rs3114020 | Tibetan: not significant | Detected only in Tibetans; not significant after correction |
| rs2231142 | Han: highly significant | Strongest functional SNP in Han |
| rs4148152 | Han: highly significant | Asian-specific locus |
| rs4148155  rs2622621 | Han: highly significant | Strongly associated locus in Han |

**Table S2. Summary of evidence strength and key findings**

| **Evidence Classification** | **Key Findings** |
| --- | --- |
| **Clinically established findings**  (Multiple large studies, consistent results, low risk of bias) | **Influencing factors:**   - Gender: Males have significantly higher HUA prevalence than females across all altitude ranges - Age: HUA prevalence decreases with age in males but increases in females ≥40 years. - Ethnicity: Han migrants have substantially higher HUA prevalence than native Tibetans at the same altitude. - Polycythemia: High‑altitude polycythemia (HAPC) is an independent and strong driver of HUA via increased purine turnover from erythrocyte production. - Renal excretion: Reduced renal UA excretion at altitude is linked to decreased renal blood flow, lower GFR, and lactate competition at proximal tubules.   **Metabolism:**   - Uric acid accounts for approximately two-thirds of total plasma antioxidant capacity.   **Disease associations:**   - HUA independently associates with albuminuria in high‑altitude populations. - HUA is markedly elevated in patients with high‑altitude pulmonary edema (HAPE) and cerebral edema (HACE). - Maternal HUA at altitude is inversely correlated with fetal birth weight, especially in proteinuria and female fetuses. - In high‑altitude indigenous children, elevated UA is positively associated with prehypertension and hypertension. - HUA is a risk factor for pulmonary hypertension and pulmonary embolism at altitude.   **Public health:**   - High‑altitude populations have substantially higher HUA prevalence than lowland populations. |
| **Emerging findings requiring validation**  (Pilot studies, single cohorts, moderate sample, plausible mechanisms) | **Influencing factors:**   - Temperature: A 5°C decrease in ambient temperature is associated with a 2.85% increase in SUA. - Dietary factors: High‑purine Tibetan diet (meat, barley wine) is associated with elevated SUA.   **Metabolism:**   - Hypoxia activates xanthine oxidase (XOD) and adenosine deaminase (ADA), accelerating purine catabolism to UA. - Short‑term high‑altitude exposure (12 months) shows increasing SUA trend; long‑term residence (>5 years) shows metabolic reprogramming toward lower SUA.   **Gut microbiota:**   - Intestinal uric acid degradation by Lachnospiraceae increases during high‑altitude adaptation and may lower systemic UA load. - Han gut microbiota shifts toward a Tibetan‑like composition with prolonged high‑altitude residence.   **Treatment:**   - Oral vitamin C (500 mg/day) reduces SUA and HUA incidence in young male military recruits at altitude. - Therapeutic erythrocytapheresis reduces SUA and improves eGFR in HAPC‑associated HUA. - β‑Alanine negatively correlates with SUA and inhibits XOD activity; potential adjunctive therapy. |
| **Exploratory / hypothesis‑generating findings** (Small samples, animal models, no independent replication) | **Influencing factors:**   - Genetic variants: Specific SNPs (ABCG2 rs3114018, WDR1 rs3756230, CLNK, PKD2) are associated with SUA, glucose, and HDL‑C in Tibetan gout patients (no replication). - Sex‑specific mechanisms: Stronger correlation of SUA with RBC in males vs. WBC in females at altitude; underlying genetic basis unexplored.   **Metabolism:**   - Metabolomic/proteomic signatures (β‑alanine, cytosine, IGLC2, C4BPA) differentiate high‑altitude HUA from controls. - Diosgenin ameliorates high‑altitude HUA in rats via EPHX2‑mediated fatty acid regulation in kidneys.   **Animal models:**   - Chronic intermittent hypoxia (CIH) activates TLR/MyD88/NF‑κB pathway, induces oxidative stress, and causes renal and hepatic injury. - CIH downregulates renal OAT1 and OCT1 expression, reducing UA secretion rather than enhancing reabsorption. - Hypobaric hypoxia reduces Na⁺,K⁺‑ATPase activity in kidneys, potentially impairing GFR and tubular function. - Fecal microbiota transplantation from plateau zokors alters gut microbiome and reduces UA in rats.   **Disease associations:**   - Serum UA change may distinguish acute mountain sickness (AMS) from non‑AMS after acute ascent. - U‑shaped relationship between maternal SUA and male birth weight in women with proteinuria at altitude.   **Treatment:**   - Inosine serves as an energy source under hypoxia and may be a therapeutic target for high‑altitude adaptation. |
